# Multisystem inflammatory syndrome in children during the first two years of the COVID-19 pandemic in Luxembourg

**DOI:** 10.3389/fped.2023.1141074

**Published:** 2023-04-05

**Authors:** C. Ooms, J. Mossong, A. Vergison, A. Biver, K. Wagner, O. Niel, A. Parrish, T. T. Abdelrahman, I. de la Fuente Garcia

**Affiliations:** ^1^ Clinique Pédiatrique, National Center for Paediatrics, Centre Hospitalier Luxembourg, Luxembourg, Luxembourg; ^2^ Department of Paediatrics, Université Catholique de Louvain, Brussels, Belgium; ^3^ Health Directorate, Strassen, Luxembourg; ^4^ Department of Microbiology, Laboratoire National de Santé, Dudelange, Luxembourg

**Keywords:** MIS-C, Luxembourg, incidence, prognosis, SARS-CoV-2 variants of concern

## Abstract

**Objective:**

Estimate the incidence of multisystem inflammatory syndrome (MIS-C) in children (0–15 years), the role of SARS-CoV-2 variants during the first two years of COVID-19 pandemic in Luxembourg; and describe the demographic, biological and clinical characteristics of the patients.

**Method:**

Observational retrospective cohort study. Cases between March 2020 and February 2022 were ascertained from the national registry of MIS-C cases by a retrospective review of medical records. Reported SARS-CoV-2 infections were obtained from the national COVID-19 surveillance system. We calculated monthly MIS-C incidence, the ratio between MIS-C and SARS-CoV-2 infections and associated rate ratios by the periods corresponding to the circulation of different variants.

**Results:**

18 children were diagnosed with MIS-C among 35,200 reported infections. The incidence rate of MIS-C was 7.2 [95% confidence interval (CI) 4.5–11.4] per 1,000,000 person-months. A higher incidence of MIS-C was observed between September and December 2021, corresponding to the circulation of the Delta variant than during the first year of the pandemic (RR 3.6, 95% CI, 1.1–12.3). The lowest rate of MIS-C per infection was observed during the Omicron (RR 0.17, 95% CI, 0.03–0.82). Median age at diagnosis was 6.5 years. Previously healthy children made up 88% of MIS-C cases, none were vaccinated against SARS-CoV-2. 33% required intensive care. All patients recovered fully.

**Conclusions:**

MIS-C incidence and MIS-C risk per infection changed significantly over time during the first two years of COVID-19 pandemic. Monitoring of MIS-C incidence in future SARS-CoV-2 waves will be essential to guide public health interventions and vaccination policies for children.

## Introduction

SARS-CoV-2 infection, a novel coronavirus which emerged in early 2020 and is responsible for the current pandemic, has been shown to affect children and adolescents less severely than adults. Most children with SARS-CoV-2 infection have mild respiratory symptoms (1).

However, a new severe paediatric condition, known as multisystem inflammatory syndrome in children (MIS-C) is a postinfectious hyperinflammatory condition that generally occurs 2–6 weeks after a typically mild or asymptomatic infection with SARS-CoV-2 (2). It was first described a few weeks after the start of the COVID-19 pandemic and constitutes one of the most serious complications of SARS-CoV-2 infection in children. This unusual inflammatory disease has now been reported worldwide. It is manifested by different clinical symptoms like high persisting fever, skin rash, multiple organ involvement and dysfunction including heart failure (3–7). Since first MIS-C cases were published, different scientific societies [Center for Disease Control and Prevention (CDC), Royal College of Pediatrics and Child Health…] have developed diagnostic criteria and case definitions. In a systematic review of 35 papers with cohorts from Europe, USA and Asia including 783 MIS-C cases 68% of the cases had to be admitted to intensive care, 77% needed cardiovascular support (fluid resuscitation or inotropic support) and 1.5% of them (12 children) died of the condition (3).

Given that MIS-C is a novel and a rare complication of SARS-CoV-2 infection, epidemiological information including incidence is relatively limited. Furthermore, incidence estimates could vary from one country to another, depending on screening strategies, genetic predisposition of the population (8), different SARS-CoV-2 variants, population immunity and SARS-CoV-2 infection incidence.

As MIS-C is one of the most serious complications of SARS-CoV-2 infection in children, it is important to monitor the incidence in order to assess the effectiveness of different public health interventions, such as vaccination, in reducing the number of cases.

### Objective

The main objective of this study was to estimate the incidence rate of MIS-C overall and during periods where different variants of SARS-CoV-2 were predominating. The secondary objective was to describe the demographic, biological, clinical characteristics as well as outcomes.

This will enable a comparison of the future evolution of MIS-C cases with the arrival of new variants and vaccination against SARS-CoV-2 in children.

## Methods

### Patient selection and MIS-C case definition

In Luxembourg, since the beginning of the pandemic, all MIS-C cases in children aged 0 to 15 years were hospitalised in the paediatric ward of a single hospital (national center for paediatrics). Since the identification of the first case of MIS-C in Luxembourg (9), a registry (electronic chart with standardized data collection) including cases of MIS-C was created in our reference center, where data from all paediatric cases were centralised. All patients aged 0 to 15 years who met the case definition for MIS-C according to CDC (10) and were admitted to our national center for paediatrics between March 2020 and the end of February 2022 were included. Patients aged between 16 and 21 years old (also included in MIS-C definition by CDC) were not included as they were not hospitalised in our paediatric center. The following data were included in the registry: demographic (age, gender, presence of comorbidities, length of hospital stay); clinical (fever, mucocutaneous, cardiovascular, neurological and respiratory symptoms), paraclinical [radiological and laboratory findings including SARS-CoV2 serology, PCR, Troponin and ProBNP (Brain Natriuretic Peptide) levels]; treatment (including immunomodulatory treatments, respiratory and cardiovascular support, intensive care admission) and outcome (recovery vs. death, organ failure and cardiac function at discharge). Circulatory failure was defined as cardiogenic low blood pressure needing inotropic support or volume resuscitation. When available, data concerning latency period (time lapse in days) between acute SARS-CoV-2 infection (first day of symptoms or positive PCR /antigenic test) and MIS-C diagnosis at hospital admission was recorded. Vaccination status against SARS-CoV-2 was also included. Patients were included in the registry during hospital admission. Data collection occurred prospectively during hospital stay or retrospectively by study investigators in charge of the patients reviewing medical files. The persons carrying out the statistical analysis herein did not have personal or identifiable data.

### National SARS-CoV-2 infections and variants

The number of laboratory reported SARS-CoV-2 infections were taken from the national COVID-19 surveillance system set up by a specific law for contact tracing activities. Different time periods where defined based on the predominating circulating SARS-CoV-2 variants at the time (>50% of samples); variants were determined by whole genome sequencing by the National Health Laboratory of SARS-CoV-2 infection data in Luxembourg Health (LNS) (https://lns.lu/en/real-time-genomic-surveillance/). Testing capacity was particularly high in Luxembourg during the whole study period with mass screening programme in place since may 2020 providing additional testing capacity for screening asymptomatic cases including children (11). Furthermore, systematic school testing (with antigenic tests) was introduced in 2021 and persisted during the whole study period. In addition to mass screening, people with symptoms and/or positive antigenic tests were able to get free PCR; and contact of cases (including contact cases in school settings and children) had the recommendation to get a free PCR in the days following the contact.

### Statistics

Incidences per study period corresponding to predominating variants and rate ratios were calculated in Stata 16.1 (College Station, USA) using a denominator of 109,387 children between 0 and 15 years old in Luxembourg (12). The demographic, biological, clinical characteristics and outcome of the different reported cases were calculated in terms of means, percentages, minimums and maximums.

## Results

**Figure 1:**
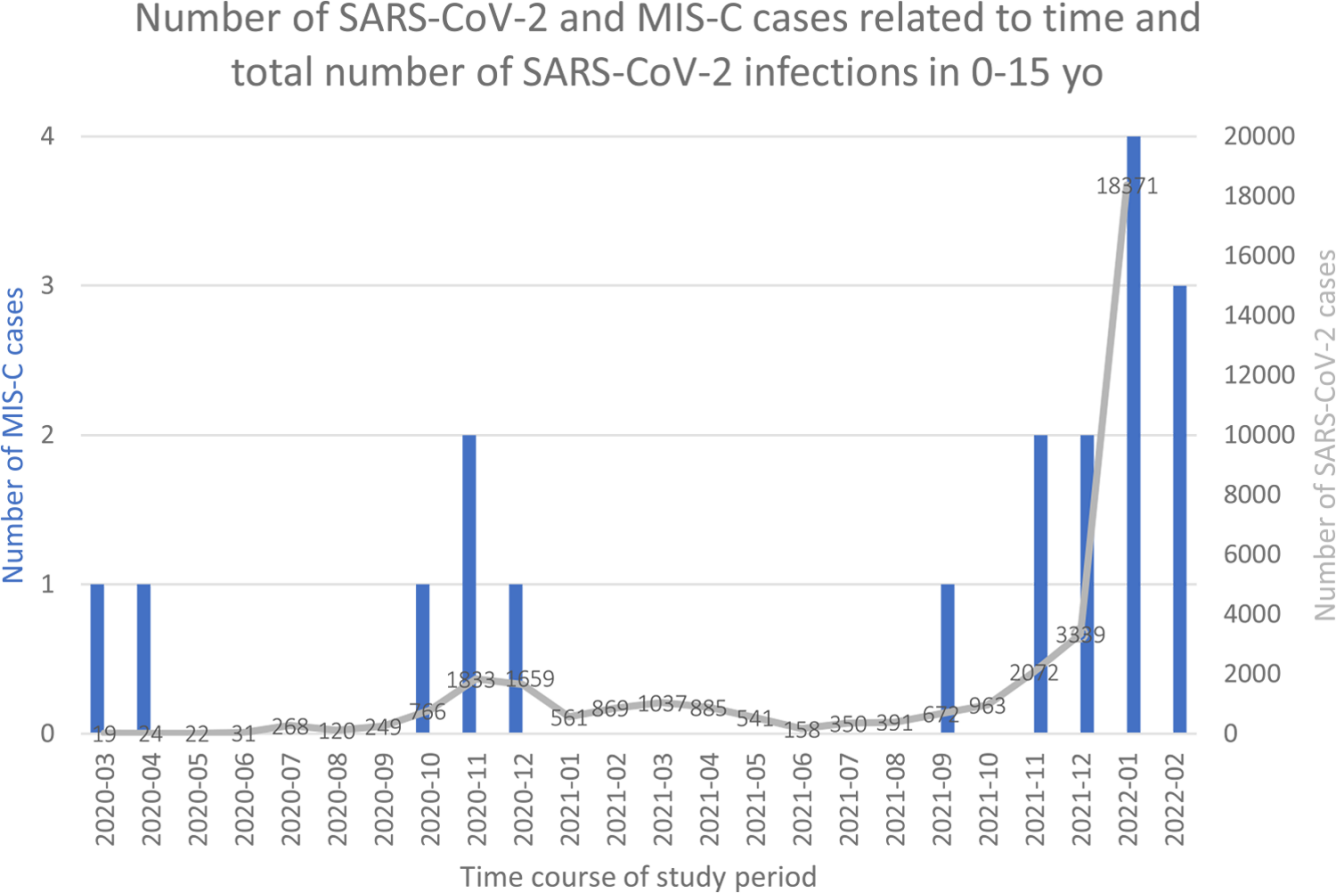
Number of SARS-CoV-2 and MIS-C cases related to time. Histograms represent number of MIS-C cases. Grey line and numbers number of SAR-coV-2 infections.

Between March 2020 and the end of February 2022, 18 patients were admitted with a diagnosis of MIS-C and a total of 35,200 laboratory SARS-CoV-2 infections were reported. This corresponds to a monthly incidence rate of MIS-C of 7.2 [95% confidence interval (CI) 4.5–11.4] per million person-months and of 51 cases per 100,000 SARS-CoV-2 infected children (or 1 per 1956 infections). As Figure 1 shows, the incidence of MIS-C cases, both relative to the population and relative to infections, changed significantly during the 2-year period.

**Figure 2:**
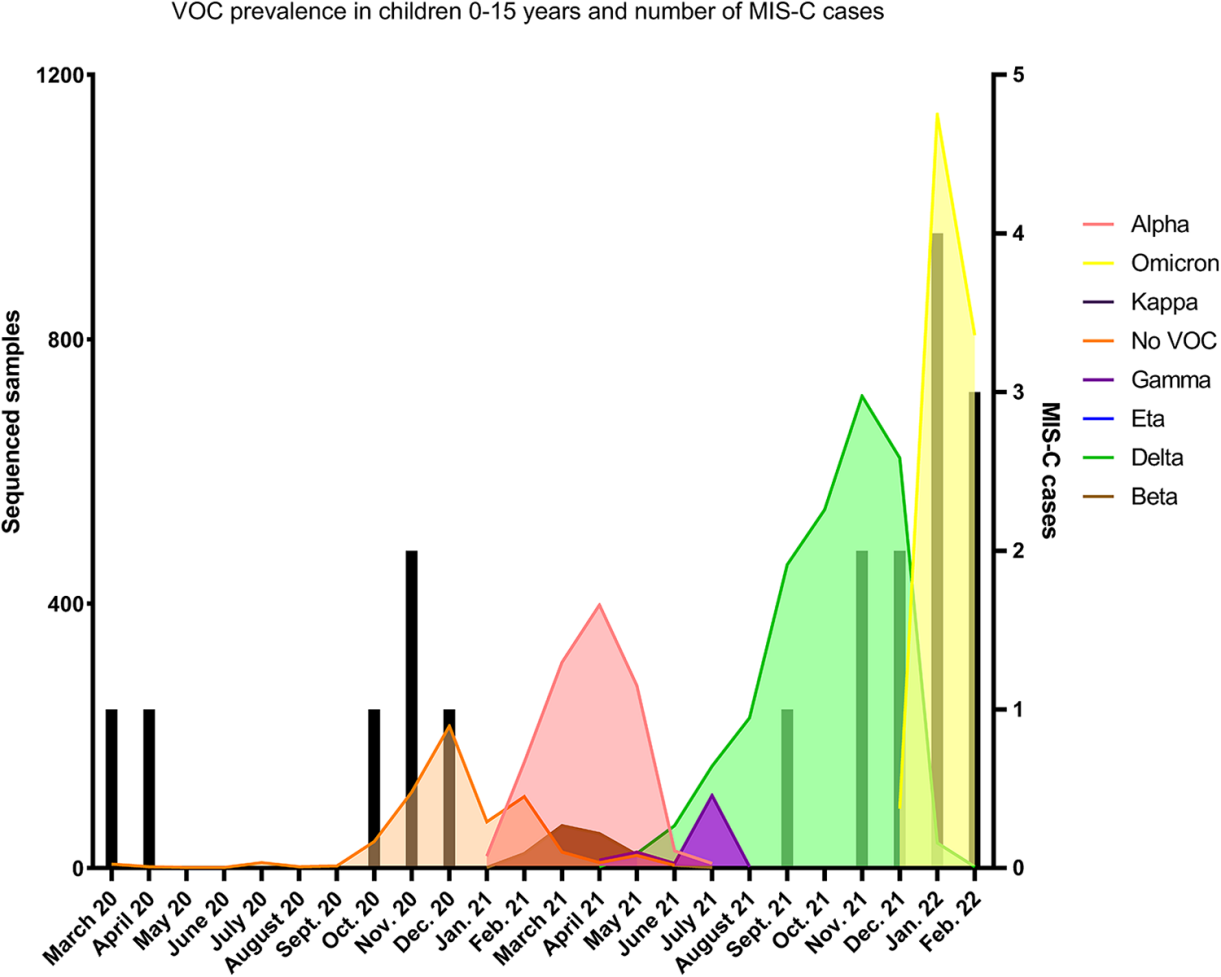
SARS-CoV-2 infections and MIS-C cases during time course of study period and variant prevalence in children 0–15. Black bars represent number of MIS-C cases and noted based on time MIS-C diagnosis. Data representative of all sequenced samples from children 0–15 years old from March 2020–March 2022. Pangolin lineage was used to assign VOCs and VOIs according to WHO definitions. No VOC represents sub lineages of original circulating strains of SARS-CoV-2.

LNS sequenced on average 55.5% of cases in 2021, allowing for accurate variant surveillance among the population. During the first year (March 2020 to February 2021) corresponding to the circulation of lineage and sublineages of the original Wuhan strain—before the arrival of variants of concern (VOC)-, the monthly incidence of MIS-C was 4.6 per million person-months (95% CI, 2.1–10.2) (see Table 1). No MIS-C cases were recorded during a period where Alpha, Beta and Gamma circulated (March until July 2021), while a rapid increase of MIS-C cases was observed during the Delta and Omicron periods (September 2021 to January 2022) (see Figure 2). The incidence rate of MIS-C cases was significantly higher during the Delta period with a relative rate of 3.6 (95% CI, 1.1–12.3) compared to the Wuhan period. After the arrival of the Omicron variant, when SARS-CoV-2 infections exploded, the case per infection rate ratio dropped significantly to 0.17 (95% CI, 0.03–0.82).

**Table 1:** Number of MIS-C cases, person months of observations, monthly MIS-C incidence, SARS-CoV-2 infections in children aged 0–15 years, rates of MIS-C cases per infection and relative rate ratios.

	Wuhan period Mar. 20–Feb. 21^a^	Delta period Aug. 21–Dec. 21^a^	Omicron period Jan. 22^a^	Whole study period Mar. 20–Jan. 22^a^
Number of MIS-C cases	6	9	3	18
Person-months	1,312,644	546,935	109,387	2,515,901
MIS-C incidence rate (per million person-months)	4.6 (95% CI, 2.1–10.2)	16.5 (95% CI, 8.6–31.6)	27.4 (95% CI, 8.8–85.0)	7.2 (95% CI, 4.5–11.4)
Incidence rate ratio (Wuhan as reference)	1	3.6 (95% CI, 1.1–12.3)	6 (95% CI, .97–28)	–
SARS-COV-2 infections	6421	7437	18,371	35,200
Rate of MIS-C cases per infection	1 per 1070	1 per 826	1 per 6123	1 per 1956
Case per infection rate ratio (Wuhan as reference)	1	1.30 (95% CI, 0.41–4.42)	0.17 (95% CI, 0.03–0.82)	–

The population aged 0–15 years included 109,387 children throughout the study period.

^a^ The periods correspond to the months of SARS-CoV-2 infections. The corresponding period for MIS-C cases are delayed by one month.

The characteristics of the study population is shown Table 2. The mean age of patients was 6.5 years with a predominance of children aged 1 to 4 years and extremes ranging from 9 months to 15 years. Males were more frequently affected than females, accounting for 78% of MIS-C cases. Only 2 children in the cohort had other comorbidities (one with Addison's disease and one with obesity). No patient was previously vaccinated against SARS-CoV-2.

**Table 2:** Demographic, biological and clinical characteristics of patients with MIS-C.

Characteristics	No	%
Age		
<12 months	1/18	6
1–4 years	7/18	39
5–10 years	6/18	33
11–15 years	4/18	22
Mean age (years)	6.5	
Sex		
Male	14/18	78
Female	4/18	22
Comorbidities		
Obesity	1/18	6
Yes (Addison's disease)	1/18	7
None	16/18	88
Contact with an individual with COVID-19	4/18	22
Recent personal infection with COVID-19	14/18	78
Time between infection/contact and diagnosis of MIS-C (days)	15–42 (mean 29)	
SARS-CoV-2 serology		
Positive	16/18	89
Not tested	2/18	11
SARS-CoV-2 RT-PCR at admission for MIS-C		
Positive	2/18	12
Negative	16/18	88
Clinical features		
Fever duration (days)	4–10 (mean 6)	
Mucocutaneous symptoms		
Rash	12/17	71
Conjunctivitis	9/16	56
Mucosa inflammation	14/17	82
Gastrointestinal symptoms	18/18	100
Abdominal pain	14/18	78
Diarrhoea	5/17	30
Vomiting	5/17	30
Cardiovascular symptoms	12/18	67
Circulatory failure	6/18	33
Neurological symptoms		
Headache	5/14	36
Lethargy	13/17	77
Irritability	8/17	47
Respiratory symptoms	8/18	45
Rhinorrhea	5/18	28
Cough	6/18	33
Acute respiratory distress (polypnea, wheezing)	1/18	5
Other		
Adenopathies	10/17	59
Oedema of the extremities	5/17	30
Image findings		
Chest radiography/computed tomography		
Normal	7/12	58
Pneumopathy	5/12	42
Cardiac echography/MRI		
Normal	7/18	39
Myocarditis	6/18	33
Coronary artery dilatation	4/18	22
Mitral insufficiency	6/18	33
Left ventricular dysfunction	4/18	22
Abdominal echography		
Normal	3/9	33
Abnormal (adenopathy, ascites, ileitis, peritonitis)	6/9	67
Treatment		
Immunoglobulin		
Single dose	12/18	67
2 doses	6/18	33
Corticosteroids	9/18	50
Acetylsalicylic acid	17/18	94
Anticoagulation	6/18	33
Antibiotics	9/18	50
Other		
Tocilizumab	2/18	11
Remdesivir	1/18	6
Diuretics	5/18	28
Outcome		
Respiratory support		
Oxygen	5/18	28
Assisted ventilation	0/18	0
Vasopressor or inotropic support	3/18	17
PICU (length of stay 2–4 days, nean 3)	6/18	33
Length of stay (days) 3–10 (mean 6)		
Death	0/18	0
Complete recovery (at the time of discharge)	18/18	100

Values presented in numbers, means and percentages, means.

PICU, paediatric intensive care unit.

The average latency period between personal infection with SARS-CoV-2 (78% of cases) or contact with a sick person (22% of cases) and the onset of MIS-C was 29 days (15 to 42 days). For all children, SARS-CoV-2 infection was recorded as their first COVID-19 episode with no prior SARS-CoV-2 infections. At the time of MIS-C diagnosis at hospital admission, serology was positive for SARS-CoV-2 antibodies in 89% of cases while RT-PCR was negative in the majority of patients (88%).

Cardiac involvement was predominant, with 6 patients showing circulatory failure (6/18, 33%) Cardiac function markers were disturbed in (16/18) 89% of cases as characterized by elevated troponin and NT pro-BNP. Cardiac ultrasound revealed various conditions such as myocarditis (6/18, 33%), coronary artery dilatation (4/18, 22%), mitral insufficiency (6/18, 33%) or left ventricular dysfunction (4/18 22%). It was found to be normal in (7/18) 39% of cases.

Other manifestations included gastrointestinal severe complications such as mimicking of acute appendicitis (5%, 1/18 cases) or aseptic peritonitis (5%, 1/18 cases). 78%% of the patients (14/18) complained of severe abdominal pain and amongst those who had an abdominal ultrasound performed (50%, 9/18) 6/9 (67%) had pathological abdominal findings (ascites, adenomegaly, ileitis.). Two patients presented with a meningeal syndrome, and one patient had acute respiratory failure, however in the context of co-infection with respiratory syncytial virus. (Table 2).

6 patients (33%) had circulatory failure and needed intensive care for cardiac support. Treatment consisted of immunomodulatory drugs in 100% of the patients (100% received immunoglobulins (2 g/kg) amongst 33% (6/18) received two doses (given in case of persisting fever after first dose), and 50% (9/18) also received steroids (given in most patients with cardiac involvement and all patients with coronary artery dilatation). Most patients (94%, 17/18) received antiaggregant therapy (low dose of acetylsalicylic acid). No patient died and 100% recovered fully with normalisation of cardiac function at cardiac ultrasound, and absence of organ failure at discharge. The mean hospitalization stay was of 6 days (Table 2).

## Discussion

Our study provides additional evidence on the occurrence and risk of MIS-C complications.

Estimation of MIS-C incidence in relation to SARS-CoV-2 infections is challenging as SARS-CoV-2 infections in children are often asymptomatic and many countries do not systematically collect data on MIS-C, e.g., in registries.

Our study estimated a relative of 1 MIS-C case in 1956 SARS-CoV-2 infections in children aged 0–15 years old. The peak in incidence of MIS-C cases was recorded between September 2021 and February 2022, a period reflecting dominance of both the delta and omicron variants in Luxembourg.

Although a limitation of our study is the relatively small size of our population leading to a small sample of patients with MIS-C during the study period, centralisation of all MIS-C cases in a single paediatric hospital (with standardised patient management and data collection), high SARS-CoV-2 testing rate in children during the whole study period and high national sequencing coverage gave us an excellent opportunity to estimate the incidence of MIS-C in Luxembourg during the first two years of COVID-19 pandemic. National policy allowed for high detection rates of SARS-CoV-2 infection through measures such as RT-PCR testing of all symptomatic cases, intense contact tracing strategies with an initial focus on schools, large scale national testing and regular school testing (primary and secondary school) through nasal antigenic tests performed one to three times per week from 2021 and until the end of the study period. These policies allowed Luxembourg to ascertain a high fraction of all cases.

Our findings are in accordance with other countries showing that MIS-C remains a rare complication of SARS-CoV-2 infection (generally <1 MIS-C case per 1,000 SARS-CoV-2 infections in children). Incidence of MIS-C cases in Luxembourg is close to the incidence reported in Sweden (estimated incidence of 6.8 cases per 100 000 persons-years during the first 10 months of the pandemic) (13) and Germany (incidence rate of 1 case per 1,357 infections in 2021) (14), but higher than that reported in southern Europe (1 per 3,700 infections in Catalonia during 2-year period) (15). Although multiple factors can contribute to explain different incidence estimations of MIS-C between different geographic regions [for example differences in local screening strategies and testing capacity, in local detection and reporting of cases, and in genetic predisposition of the population in accordance to ethnic origin (8)], an important factor that could play an important role in explaining MIS-C incidence differences between different regions over time could be differences in population immunity throughout the pandemic. As SARS-CoV-2 circulation and infection prevalence was heterogeneous from country to country particularly during the first months of the pandemic; and as previous SARS-CoV-2 immunity is probably a protective factor against development of post infectious complications from subsequent SARS-CoV-2 infections, countries with a high population (including in children) immunity due to high prevalence of infection during the first waves of the pandemic, had possibly a protective effect against MIS-C at the arrival of subsequent virulent SARS-CoV-2 waves including Delta and Omicron waves. Further studies from different countries and further data analysis of MISC incidence accordingly to prior population immunity will be needed to confirm this hypothesis.

In Luxembourg, high incidence of MIS-C was associated with circulation of the Delta variant compared to other VOCs. Omicron infection had a lower relative risk of MIS-C development, however given the increased number of infections in children with this variant, total numbers of MIS-C during that period remained high. An important finding is that none of the patients with MIS-C diagnosis in this study had prior immunity against SARS-CoV-2 (neither by natural infection nor by vaccination) supporting the hypothesis that naïve children to SARS-CoV-2 (by infection or vaccination) are probably at higher risk of post infectious complications like MIS-C after infection.

Data on MIS-C rate risk accordingly to different SARS-CoV-2 variants is scarce and heterogeneous from one country to the other with little surveillance and reliable data during the first pandemic year (as most countries performed low testing rates in children). However, reduction of risk associated with omicron circulation has consistently been described in other countries (UK, Germany, Denmark, Spain and Australia) (15–19) like in Luxembourg giving promising perspectives for the near future as with increased population immunity (natural and vaccination immunity) and current circulating VOC we can expect MIS-C incidence to remain low. A few recent studies have indeed shown that vaccination against COVID-19 was associated with a high level of protection against MIS-C (18, 20).

Unfortunately, we lack information regarding SARS-CoV-2 variant that caused each MIS-C episode, nevertheless all variants achieved prevalence rates >90% supporting the relation between the infection and the circulating VOC.

Although the diagnosis of MIS-C can be challenging as there is no definitive diagnostic test for MIS-C diagnosis, and clinical and paraclinical features overlap with Kawasaki disease (KD), these two syndromes have important specific and distinct characteristics that have already been highlighted in previous publications: MIS-C affects older children in contrast with KD (peak incidence at 9–11 months of age with affected children being generally <5 years of age) (21), abdominal manifestations are rare in KD but common in MIS-C patients and myocardial dysfunction manifest in a higher incidence of patients with MIS-C than with KD (22). These features have also been emphasized in our cohort, with a mean age of our patients being 6,5 years old, and a very high proportion of cases showing gastrointestinal manifestations (100% of patients) and cardiac anomalies on cardiac imaging studies (61%). In two different meta-analysis involving in total 1,700 MIS-C cases (3, 23) gastrointestinal symptoms occurred in 71% and 87% of the patients, and abnormal echocardiogram/myocardial dysfunction in 59% and 55% of the cases. Concerning other demographic and clinical characteristics of our national cohort, findings are also comparable to other published cohorts with most affected children being previously healthy (2, 5, 15), developing high rate of cardiac complications but evolving favourably in most of the cases with relatively short hospital stay (<10 days) (1, 5, 14, 15,). With prompt diagnosis and treatment, 100% of our patients had an excellent evolution with no permanent organ failure.

It is therefore essential to continue monitoring MIS-C incidence and assess the effectiveness of vaccination and the impact of immunity with previous SARS-CoV-2 infections in the risk of MIS-C development to guide and adapt different public health interventions and vaccination policies for children during the COVID-19 pandemic.

## Data Availability

The raw data supporting the conclusions of this article will be made available by the authors, without undue reservation.
